# A Rare Case of Early Gastric Cancer With Rapid Bone Involvement

**DOI:** 10.7759/cureus.66317

**Published:** 2024-08-06

**Authors:** Taleen A Ashikian, Manuel E Babaian

**Affiliations:** 1 Medicine, Université de Montréal, Montreal, CAN; 2 Gastroenterology, Holy Cross Hospital, Fort Lauderdale, USA

**Keywords:** thoracic trans-hiatal esophagogastrostomy, early gastric cancer diagnosis, gastric cancer screening, bone metastasis, gastric cancer

## Abstract

Gastric cancers rarely metastasize to the bones. If they do, they have a very poor prognosis. We here present a case study of a 56-year-old man who, within a year, rapidly declined and died. He was first revealed to have an erosion found on an esophageal gastroduodenoscopy (EGD), which was later proven to be a poorly differentiated gastric adenocarcinoma. He then proceeded to have a thoracic trans-hiatal esophagogastrostomy with gastric pull-up to resect this cancer. At this point in time, the review of systems and CT scans of the abdomen and pelvis were negative. A few months later, he started having back pain and was diagnosed with metastatic disease of the bones through a CT scan. Although detecting gastric cancer at an early stage is rare, it is shown to have a better prognosis. It is, therefore, very important to reflect on the possibility of engaging in earlier screening to detect gastric cancers at an earlier stage to minimize the risk of invasions of other organs, especially for those who have other risk factors such as obesity and tobacco use. We believe it is prudent to ensure close follow-up with any patient with early gastric cancer to potentially detect recurrence or metastasis in a timely fashion.

## Introduction

Gastric cancer is usually diagnosed at an advanced stage. It most often presents with typical symptoms of abdominal discomfort, nausea, loss of appetite, and weight loss. Often, at the time of diagnosis, it has metastasized and is, most of the time, incurable. Bone metastasis is quite rare, and if it does happen, it unfortunately has a very poor prognosis, as in our case report. It is one of the leading causes of cancer-related deaths. However, when it is diagnosed at an early stage, there might be a better clinical outcome in terms of improved quality of life and sometimes a longer survival period. More preventive efforts can be made by reducing environmental and dietary risk factors. More efforts to establish screening programs for early stomach cancers would be helpful since the highest cure rate for gastric cancer is associated with asymptomatic patients when the tumor is limited to the mucosa. This report emphasizes the need for early intervention when facing an aggressive cancer that can progress very rapidly.

## Case presentation

Initial clinical presentation

A 56-year-old man, born in the region of southern Caucasus, presented electively complaining of symptoms of indigestion, epigastric abdominal pain and intolerance to certain acidic foods. These symptoms were present for approximately six to eight weeks prior to the consultation. The review of systems was negative; in particular, there was no notable weight loss, anemia, any sign of bleeding, or back pain. However, there was a five-year history of smoking in the past. Furthermore, there was no significant history of stomach or colon cancer in the family.

**Figure 1:**
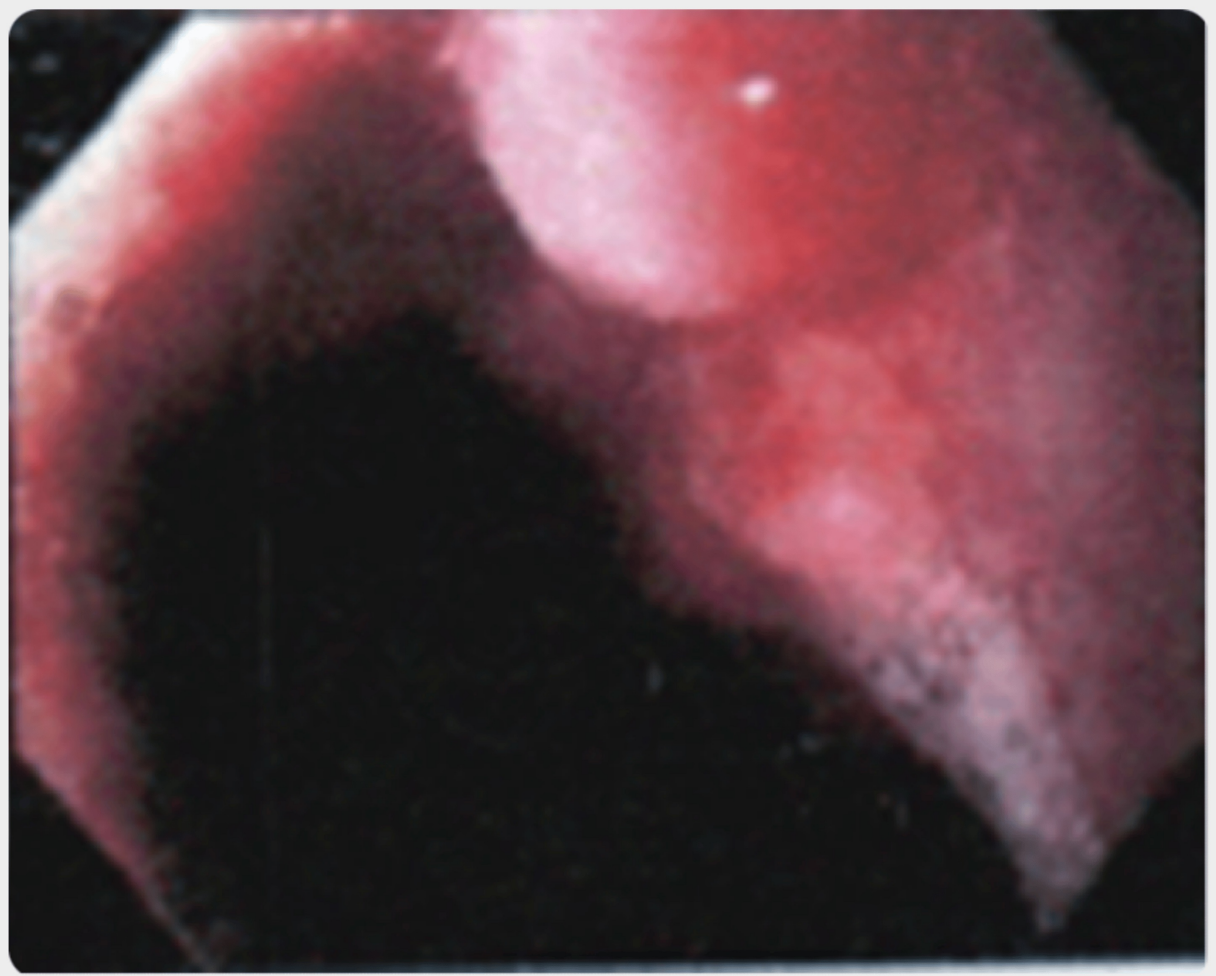
Erosion found at the cardia in esophageal gastroduodenoscopy

The patient had undergone a routine negative colonoscopy five years earlier. At the time of presentation, an esophageal gastroduodenoscopy (EGD) was performed. There was inflammation in the esophagogastric area. There was a 2 to 3-cm erosion associated with mild erythema found in the cardia portion of the stomach (Figure 1). Multiple biopsies confirmed a poorly differentiated adenocarcinoma signet cell cancer of the stomach. No Helicobacter pylori was detected. There was also LA-grade B oesophagitis. At this time, the CT scan of the abdomen and pelvis, including the lumbar spine, did not show any presence of metastasis.

Follow-up and outcome

Due to the COVID-19 pandemic, the surgery was only able to be performed three months after the diagnosis. The oncology service, in consultation with the surgeon, made a decision against neoadjuvant chemotherapy in view of the absence of metastatic disease at that time. The patient underwent a thoracic trans-hiatal esophagogastrostomy with gastric pull-up, which he tolerated well.

**Figure 2:**
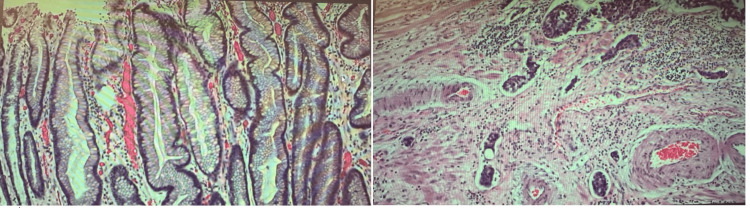
Histology slides of gastric cancer with lymphovascular invasion

The stage of the cancer, in this case, corresponded to T1aN0M0. The surgical pathology report revealed a G3 poorly differentiated adenocarcinoma, which was found at the esophagogastric junction. The size of the tumor was 3.3 cm, and it infiltrated into the muscularis propria at a depth of 90%. Histology slides revealed gastric cancer with lymphovascular invasion (Figure 2). The margins were not involved in invasive carcinoma, dysplasia, or intestinal metaplasia. There were no lymph nodes involved after the examination of 16 lymph nodes. The primary tumor corresponded to pT2, and the regional lymph nodes corresponded to pN0.

Seven months after the initial diagnosis of the poorly differentiated adenocarcinoma, the patient reported lumbar pain, for which a lumbar spine CT scan was done. The patient ultimately underwent a CT-guided biopsy, which confirmed an adenocarcinoma with signet cells at the spine. The pathology report correlated very closely with the primary gastric tissue. This indicated that the cancer found in the spine was a metastatic spread of the primary gastric one. Within the next three months, the patient unfortunately succumbed to his illness.

## Discussion

At the time of initial diagnosis, the usual symptoms of gastric adenocarcinomas include unexplained weight loss and abdominal pain [1]. Gastric adenocarcinomas usually metastasize to the liver (48%), the peritoneum (32%), and the lungs (15%) [2]. The percentage of metastasis of gastric cancer that spreads to the bones corresponds to 3.8%, according to the Journal of Gastric Cancer [3]. Patients who have bone metastasis have, unfortunately, a poorer prognosis, with a median survival period of approximately three to four months [4]. Unfortunately, gastric cancer has been identified as the third-leading cause of cancer-related deaths [5].

Early gastric cancer corresponds to a tumor that invades only the mucosa or submucosa of the stomach [6]. Early gastric cancers correspond to around 20% of gastric cancers in Western countries [6]. In contrast to Western countries, Eastern countries such as Japan engage in nationwide screening programs; therefore, their rate of early gastric cancer is superior to that of Western countries at around 50% [6]. The prognosis rate for the Eastern countries is generally superior compared to the Western ones [6].

In this patient’s case, even though the cancer was diagnosed early, only the submucosa was invaded, and surgery was performed with negative CT scan results at that time. Unfortunately, metastatic disease was unexpectedly found in the bones. When we face early gastric cancers, it is advisable to have regular follow-ups and do an appropriate diagnostic evaluation, such as ordering CBC, LFTs, and additional imaging, such as MRI, to assist in the early detection of a cancer recurrence or metastasis.

Because of multiple negative studies, including CT scans and lymph node evaluations, we did not expect metastatic disease, least of all involvement of the bones. Most of the studies on metastatic gastric cancer in bones in the literature are retrospective [7]. Further prospective studies would be helpful in categorizing patients and their risks for early recurrence and metastasis. One possibility to reduce the risk would be an earlier screening process, especially for patients with significant risk factors. In the literature, we see that many significant risk factors have been identified, such as prior Helicobacter pylori infections, an occupational risk specifically for those who work in the rubber or coal industry and/or are exposed to radiation, a diet that is considered low in fruits and vegetables and/or elevated salt intake, tobacco use, a family history such as having a first-degree relative with stomach cancer, obesity, prior Epstein-Barr viral infection, pernicious anemia, chronic atrophic gastritis and atrophic gastritis with intestinal metaplasia [8]. Furthermore, the country of origin also plays a role in risk factors for gastric cancer. Specifically, the literature suggests that people from Eastern and Central Asia have a higher tendency to develop gastric cancers [9]. Eastern countries such as Japan screen on a much earlier and more regular basis for gastric and oesophageal malignancies and the diseases are therefore diagnosed at earlier stages with a consequently superior prognosis [10].

## Conclusions

Detection of gastric cancer at an early stage is uncommon. If we find gastric cancer at an earlier stage rather than a later stage, it is associated with better clinical outcomes. Thus, it is recommended to implement screening programs for patients who have significant risk factors for the identification of early gastric cancer cases before there is a significant spread outside the stomach associated with the worst clinical outcomes. Screening should be done routinely for individuals with significant risk factors.

Metastatic spread can potentially occur rather early on, after the initial diagnosis of gastric cancer. Therefore, vigilance and close follow-up are recommended for these patients, including appropriate blood tests and imaging. This early detection would give us the opportunity to potentially modify treatments and improve the quality of life of these vulnerable patients.
